# Supplementary figures and images for: A computational workflow for assessing drug effects on temporal signaling dynamics reveals robustness in stimulus-specific NFκB signaling
Source: PLoS Comput Biol. 2025 Aug 21;21(8):e1013344. doi: 10.1371/journal.pcbi.1013344 (PMC12370059; doi:10.1371/journal.pcbi.1013344)

S1 Fig

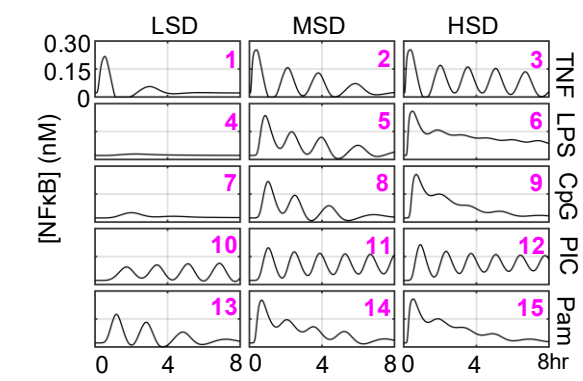

Supplement: S1 Fig — Trajectories of nuclear NFκB concentration (y-axis of each plot) over time (x-axis of each plot) for untreated conditions across 5 ligands and 3 doses. Rows indicate different ligands (labeled on the right of the panel), and columns specify ligand doses (labeled on the top of the panel). Stimulation indices are labeled in the top right corner. (PDF) [file pcbi.1013344.s002.pdf]

S2 Fig

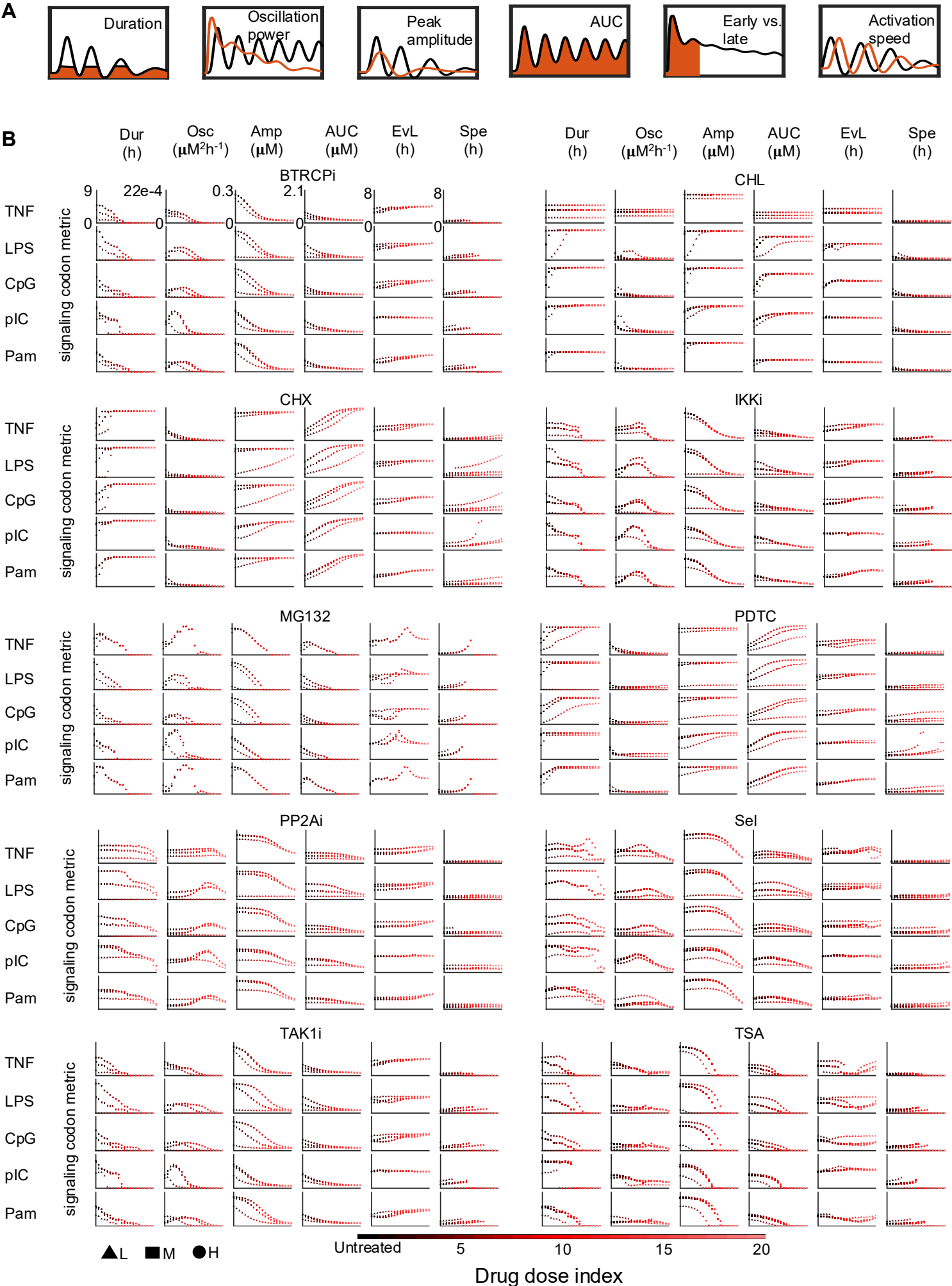

Supplement: S2 Fig — (A) Illustration of six signaling dynamic codons: Duration (Dur), Oscillations (Osc), Peak Amplitude (Amp), Total Activity (AUC), Early vs. Late Activity (EvL), and Activation Speed (Spe). (B) Signaling dynamic codons for nuclear NFκB trajectories following pharmacological perturbation. For each drug panel, signaling dynamic codons are stratified by column (labeled on the top) and stimuli are stratified by row (labeled on the left). Each individual plot pertains to one signaling codon (y-axis) for a particular stimulus at 3 doses (low – triangle, medium – square, high – circle, labeled on the bottom left), ranging from untreated to drug dose 20 (log scale x-axis). (PDF) [file pcbi.1013344.s003.pdf]

S3 Fig

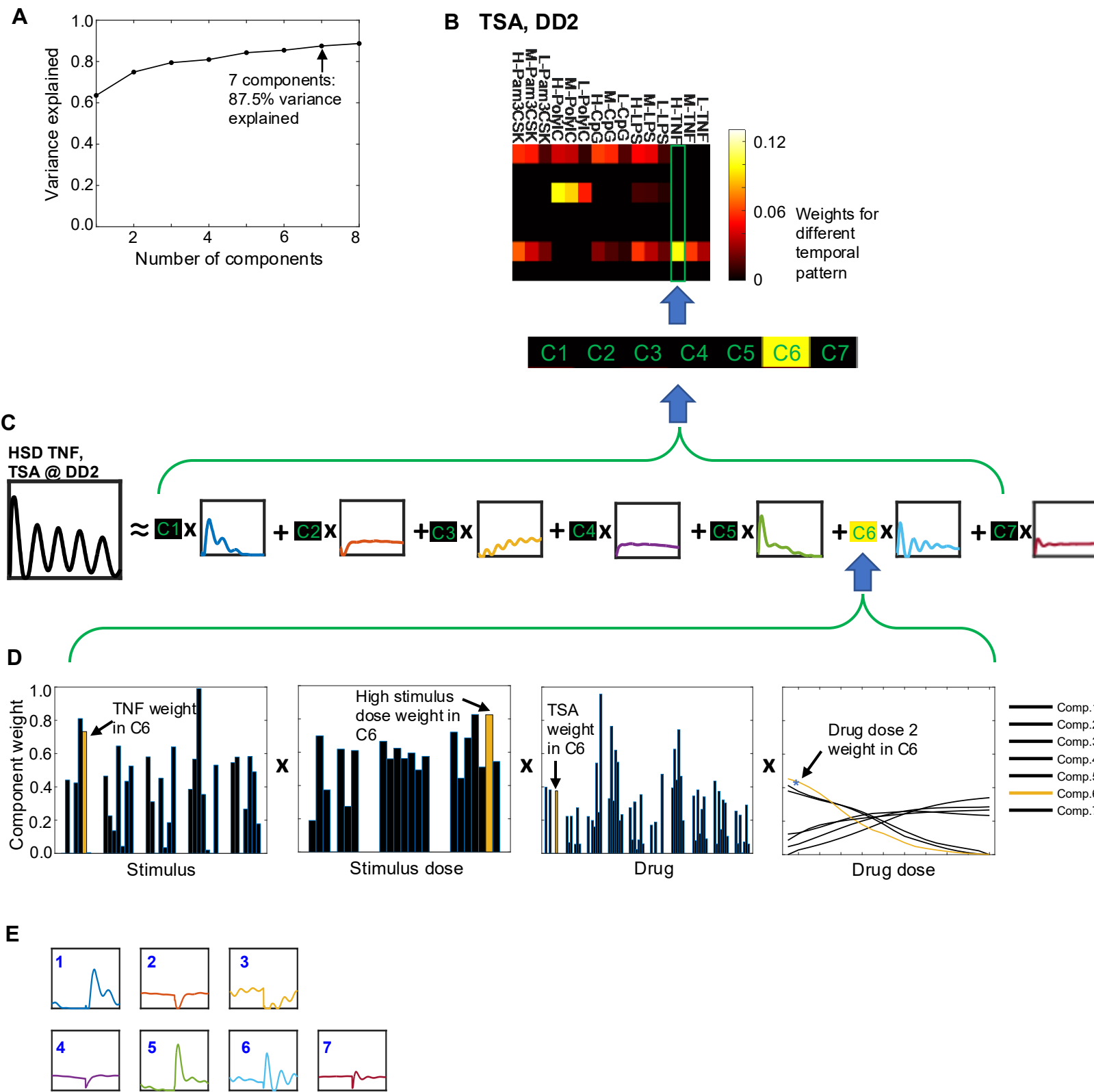

Supplement: S3 Fig — (A) R2X plot depicting the percent variance explained after the application of CP decomposition for 1–8 components. (B) Heatmap of weights corresponding to seven temporal patterns using the drug treatment TSA at drug dose (DD) 2 as an example in defining the landscape for specific drug regime. Inside the heatmap, 15 rows depict 15 stimuli (5 ligands at 3 different doses). Colors within each row indicate the weights of the seven temporal patterns. These weights are the product of the component weights associated with the respective drug, drug dose, ligand, and ligand dose dimensions. The specific order of the 15 stimuli is outlined on the top of the figure, with L, M, and H denoting low-dose, medium-dose, and high-dose respectively. The weights of the temporal pattern (squares labeled by C1-C7 represent the weights for component 1–7 temporal patterns, respectively) stimulated by high-dose TNF under the TSA DD2 drug regime are specifically highlighted. (C) An example of the high-dose TNF stimulated NFκB trajectory perturbed by TSA at DD2 is displayed on the left. This can be approximated by the weighted sum (weights indicated by colors in the squares) of the seven temporal patterns (displayed adjacent to the colored squares). The weights are the product of the component weights corresponding to the specific drug, drug dose, ligand, and ligand dose dimensions obtained from CP decomposition. (D) The weights employed in the reconstruction of the simulated trajectory are the products of the weights in the dimensions of ligand, ligand dose, drug, and drug dose, with the yellow bars providing an example of calculating the weights for component 6 of (C). The subpanels and Fig 3E are the outcomes of Canonical Polyadic Decomposition (CPD) to nuclear NFκB time trajectory tensor, resulting in seven distinct components. These components are represented by their respective weights across various dimensions: time (Fig 3E), ligand, ligand doses, drugs, and drug dose index (this figu [file pcbi.1013344.s004.pdf]

S4 Fig

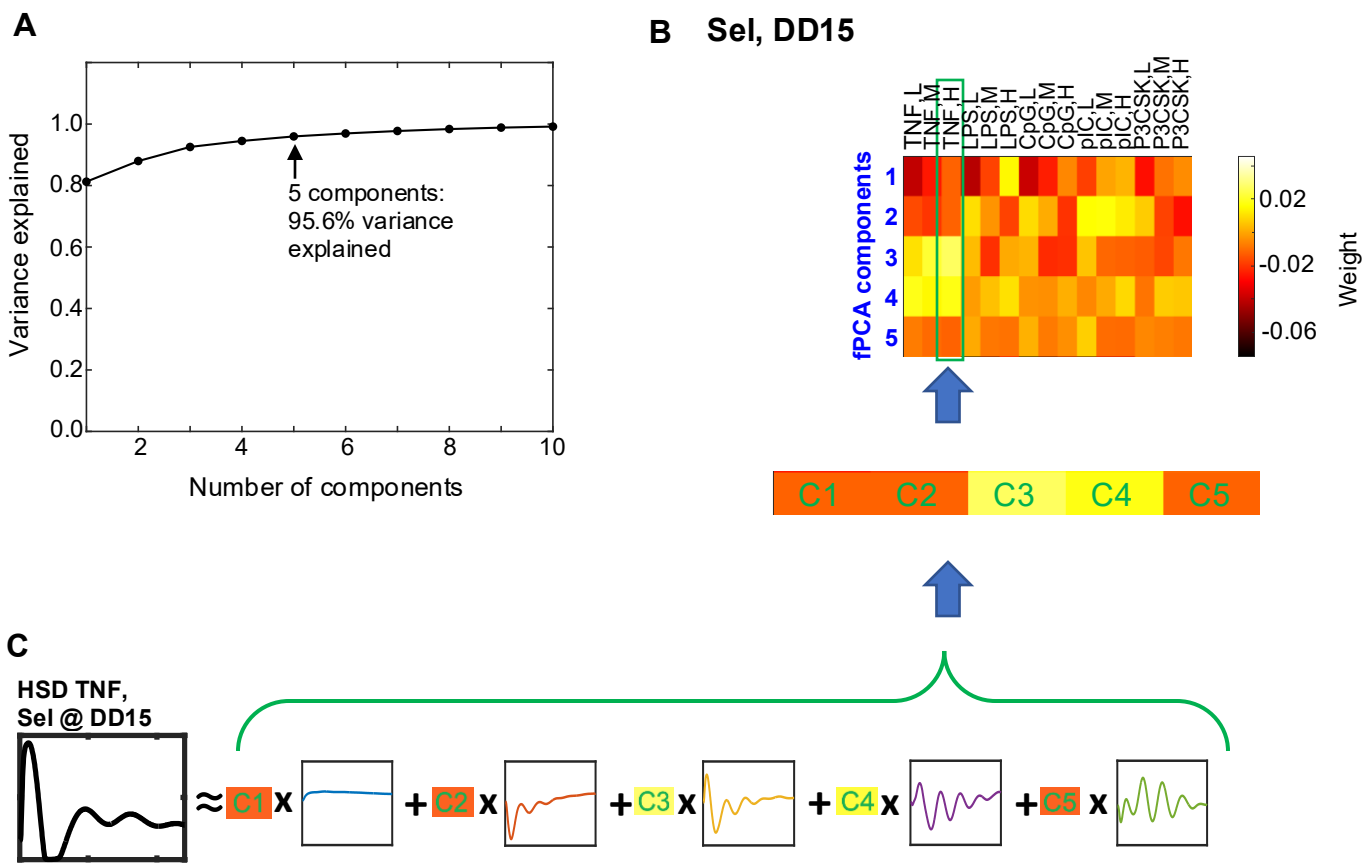

Supplement: S4 Fig — (A) R2X plot depicting the percent variance explained after applying fPCA using 1–10 principal components. (B) Heatmap of weights corresponding to five temporal patterns for the drug treatment Sel at drug dose (DD) 15. The weights are the score outputs from the decomposition. The 15 heatmap rows correspond to the 15 stimuli (5 ligands at 3 different doses) with their order annotated at the top of the heatmap (L = low-dose, M = medium-dose, H = high-dose). The squares labeled C1-C5 represent the five temporal pattern (i.e., components 1–5) weights for NFκB activity under high-dose TNF stimulation and perturbed by the Sel DD15 drug regime. (C) An example of the high-dose TNF stimulated NFκB trajectory perturbed by Sel at DD15 is displayed on the left. This can be approximated by the weighted sum (weights indicated by colors in the squares) of the five temporal patterns (displayed following each colored square). (PDF) [file pcbi.1013344.s005.pdf]

S7 Fig

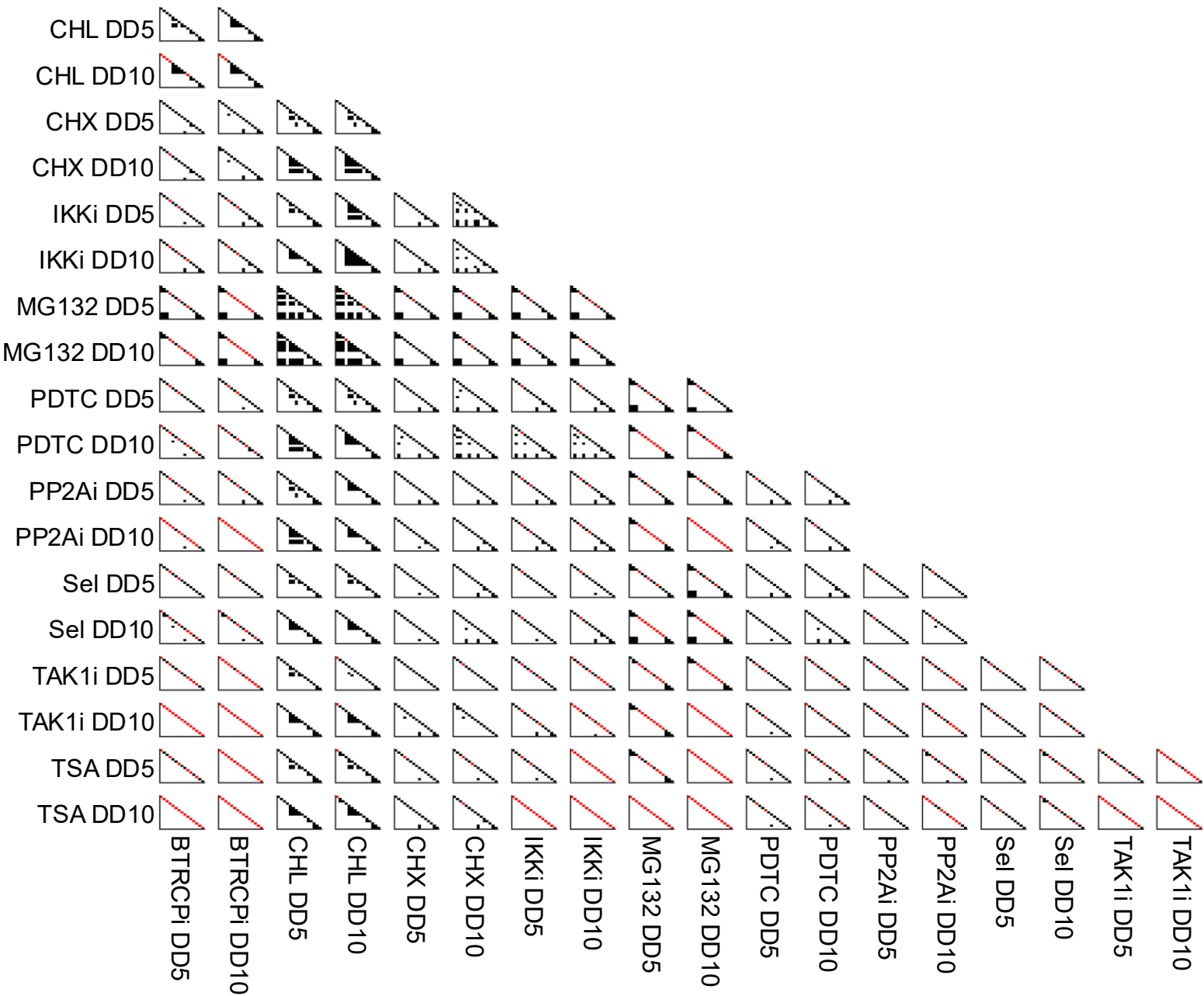

Supplement: S6 Fig — (A) Stimulus cluster maps for trajectories treated with MG132 at DD6. (B) Examples of decreased temporal coding capacity (high doses of TNF and Pam3CSK, top row) and increased temporal coding capacity (high doses of Pam3CSK and CpG, bottom row), represented in the trajectory space (left and middle columns) and signaling codon space (right column). (PDF) [file pcbi.1013344.s007.pdf]

S8 Fig

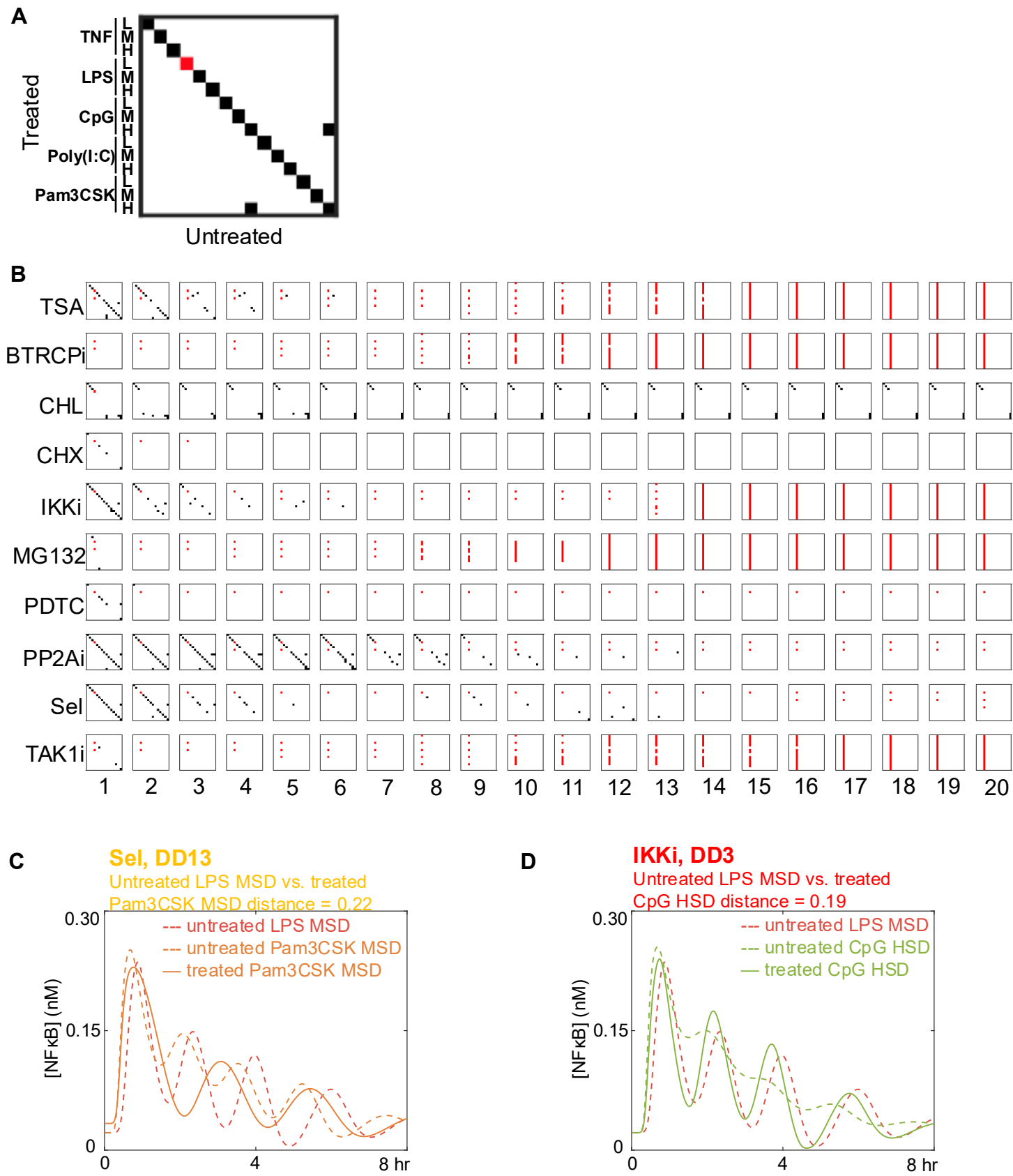

Supplement: S7 Fig — Stimulus cluster map for the 15 stimulus conditions under 180 drug combination regimes. The 180 regimes are comprised of 45 combinations of the 10 drugs and 4 dose combinations using DD5 and DD10 (45 x 4 = 180). Columns represent untreated stimuli and rows represent treated stimuli. Black clusters on and off the diagonal represent “confusion” between stimulus conditions’ NFκB responses. Red squares on the diagonal represent inhibited NFκB signaling (non-responders). (PDF) [file pcbi.1013344.s009.pdf]
