# Supplementary material for: A computational workflow for assessing drug effects on temporal signaling dynamics reveals robustness in stimulus-specific NFκB signaling
Source: PLoS Comput Biol. 2025 Aug 21;21(8):e1013344. doi: 10.1371/journal.pcbi.1013344 (PMC12370059; doi:10.1371/journal.pcbi.1013344)

S5 Fig

A

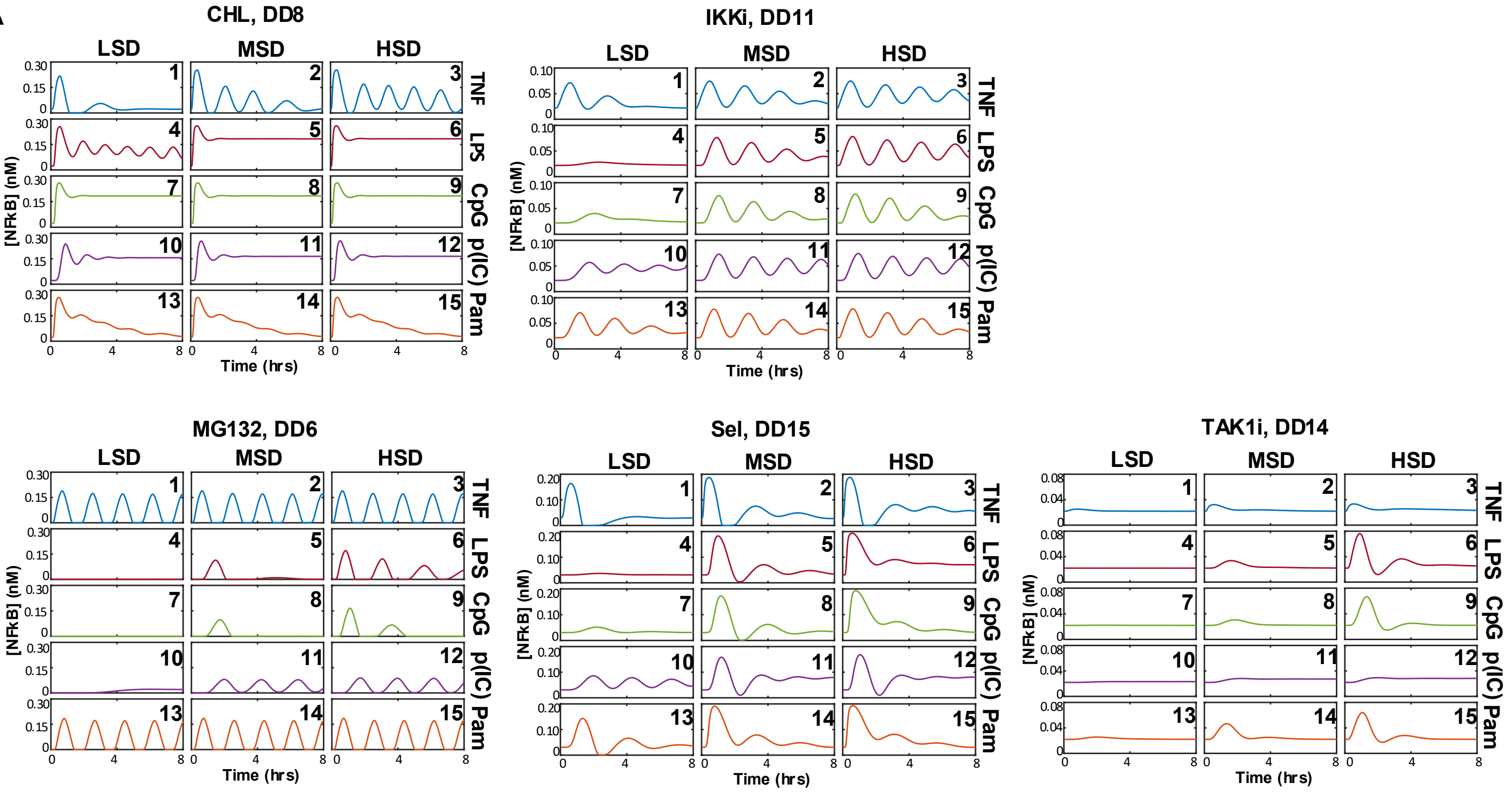

B

Drug regime	CHL, DD8	IKKi, DD11	MG132, DD6	Sel, DD15	TAK1i, DD14
Expert classification	[1],[2],[3],[4], [5,6,7,8,9], [10],[11,12], [13,14,15]	[1], [2,5,9,14,15], [3,6],[4,7], [8,13],[10], [11,12]	[1-3,13-15], [4,7,10],[5,8], [6],[9],[11,12]	[1],[2],[3],[4], [5,8],[6],[7], [9,14,15],[10], [11,12],[13]	[1-5,7,8, 10-14], [6],[9,15]

C

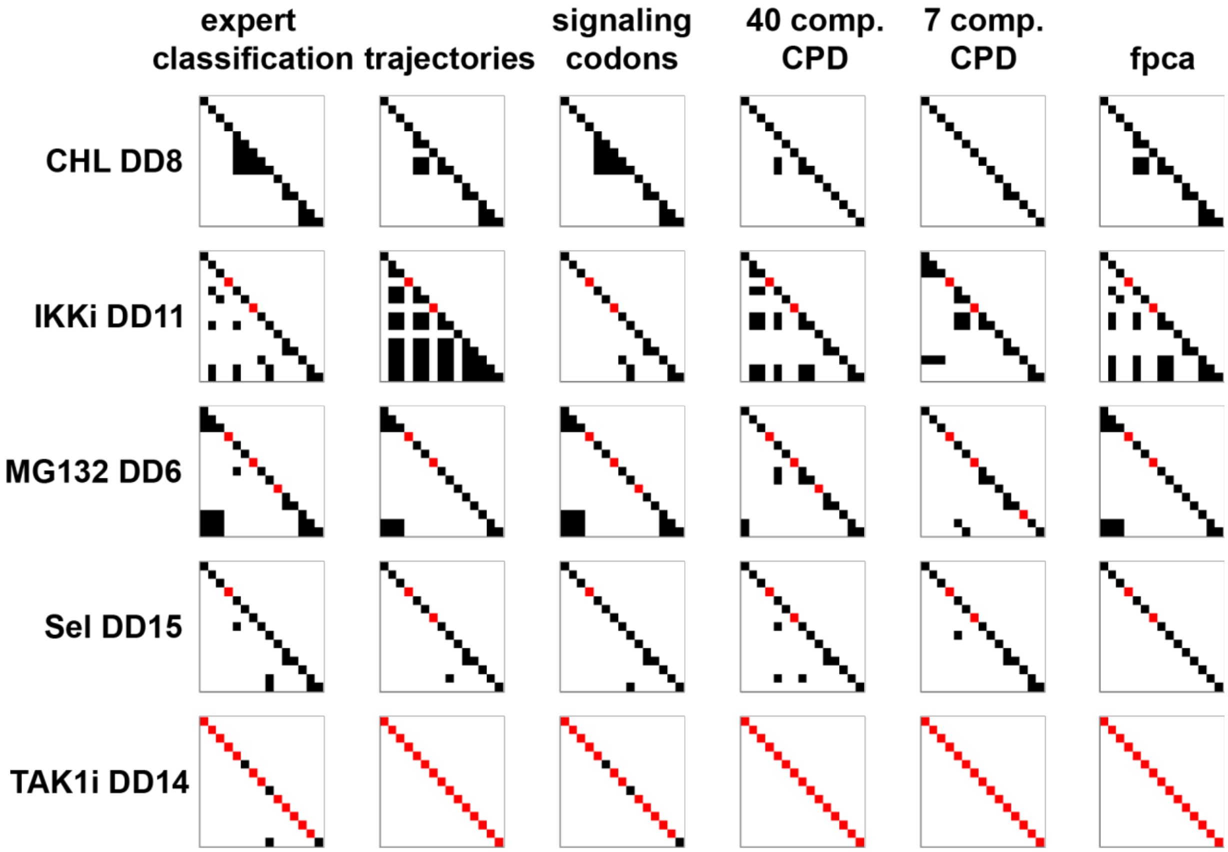

Supplement: S5 Fig — (A) Trajectories of nuclear NFκB concentration (y-axis of each plot) over time (x-axis of each plot) for drug regimes CHL DD8, IKKi DD11, MG132 DD6, Sel DD15, TAK1i DD14 across 5 ligands and 3 doses. Solid colored lines represent the trajectories under drug treatment, while dashed lines depict untreated trajectories. Rows indicate different ligands (labeled on the right of each set of drug regime plots), and columns specify ligand doses (labeled on the top of each set of plots). Stimulation indices are labeled in the top right corner. (B) Expert classifications for 5 representative regimes: CHL DD8, IKKi DD11, MG132 DD6, Sel DD15, and TAK1i DD14 used in epsilon network clustering. Clusters for each regime are annotated according to the trajectory indices in (A). (C) Stimulus cluster maps constructed from epsilon network clustering results for 5 representative regimes. Each row and column within one map corresponds to a specific stimulus, as denoted in S6A Fig. Within each map, off-diagonal black squares represent responsive clusters and red squares on the diagonal represent inhibited NFkB signaling (non-responder). Panels from left to right display the expert classifications, clusters derived from the trajectory space, signaling codon space, 7 component CPD feature space, and 40 component CPD feature space, and fPCA feature space (Labeled on the top of the panel). (PDF) [file pcbi.1013344.s006.pdf]
